# A Narrative Review of Implementing Nonpharmacological Interventions for Antenatal Depression in Low Resource Settings With Implications for Palestine

**DOI:** 10.1155/da/4692841

**Published:** 2026-08-11

**Authors:** Sawsan Saeed Abd Al Rahman Shqair, Mohammed Hayek, Loai M. Zabin, Mohammad Marie, Jamal Qaddumi, Shurouq Ghalib Qadous, Adnan L. Sarhan

**Affiliations:** ^1^ Palestinian Ministry of Health, Nablus, State of Palestine, moh.ps; ^2^ Faculty of Nursing, An Najah National University, Nablus, State of Palestine, najah.edu; ^3^ Nursing Department, Technical Community College, Al Zaytona University, Salfit, State of Palestine; ^4^ Department of Public Health, Faculty of Medicine and Applied Science, An Najah National University, Nablus, State of Palestine, najah.edu

**Keywords:** acupuncture therapy, depressive disorder, mental health services, palestine, pregnancy, psychotherapy

## Abstract

Antenatal depression affects a substantial proportion of pregnant women and is associated with adverse maternal and infant outcomes, particularly in settings where specialist mental health services are limited. The purpose of this narrative review was to synthesize evidence on the effectiveness and implementation feasibility of nonpharmacological interventions for antenatal depression in low‐resource settings, with particular attention to task sharing, cultural adaptation, and relevance to Palestine. A targeted search of PubMed, ScienceDirect, SpringerLink, JMIR Publications, Google Scholar, and an Arabic‐language journal was conducted to 30 September 2025. Eligible studies included pregnant women with depressive disorders or clinically relevant depressive symptoms, as well as prevention‐oriented antenatal samples in which depressive symptoms were a prespecified outcome. Eligible publications evaluated nonpharmacological interventions using randomized, quasi‐experimental, or prospective pre–post designs; linked reports of pregnancy or neonatal outcomes from otherwise eligible antenatal intervention programs were also considered. Twenty‐two studies met the inclusion criteria. Generally favorable findings were reported for cognitive behavioral therapy (CBT)‐ and interpersonal psychotherapy (IPT)‐based interventions delivered in individual, group, task‐shared, and brief clinic‐based formats, although the strength and consistency of evidence varied by study design and population. Favorable findings were also reported for depression‐specific acupuncture, brief mindfulness and mind–body programs, nurse‐supported counseling, and culturally or spiritually integrated approaches, although the strength of inference varied by study design. Supervised digital formats and task‐shared models involving primary health workers, nurses, and midwives showed promise for extending care in constrained systems, whereas unguided self‐help approaches produced less consistent findings. Implementation reporting was uneven: service integration, task sharing, brief delivery, and supervised formats supported preliminary feasibility, whereas formal measures of acceptability, fidelity, cost, population reach, and sustainability were infrequently reported. The review identified several potentially implementable nonpharmacological options for antenatal depression in low‐resource settings, particularly brief psychological therapies, task‐shared models, selected supervised mindfulness formats, nurse‐supported counseling, and culturally adapted programs. However, locally codesigned pragmatic studies are required before broad implementation in Palestine and similar humanitarian or resource‐constrained settings.

## 1. Introduction

Antenatal depression is common and clinically significant worldwide, with prevalence estimates typically ranging from 10% to 20% of pregnant women [1]. Burdens tend to be lower in high‐income settings (~10%–15%) and higher in economically poorer countries (19%–25%) [2]. Depression is a leading cause of disability among women of reproductive age and is more prevalent in women than in men; nearly one in four women experiences depression at some point in life [1, 3]. Pregnancy may heighten vulnerability to depressive symptoms and disorders, and untreated antenatal depression adversely affects maternal and fetal well‐being and family functioning; it is also a contributor to subsequent postnatal depression [4, 5]. Reported adverse outcomes linked to untreated perinatal depression include poor infant growth in the first year of life, preterm labor, and low birth weight [5]. Despite the public health significance of antenatal depression, much of the maternal mental health literature has historically emphasized postpartum depression, leaving antenatal depression comparatively under‐recognized in many regions [5, 6]. To maintain interpretive rigor in this review, we distinguish the descriptive burden from causal inference throughout.

Multiple treatment options exist in pregnancy, including pharmacological (antidepressants) and nonpharmacological approaches (psychological and complementary/alternative modalities) [7]. Although antidepressants can reduce symptoms for some pregnant patients [8], many women prefer nonpharmacological interventions during pregnancy [9]. Psychotherapy, particularly cognitive behavioral therapy (CBT) and interpersonal psychotherapy (IPT), is recommended as an important treatment option for antenatal depression and is widely used in clinical practice [10, 11]. CBT targets maladaptive cognitions and behaviors and may be especially useful when social stressors are prominent [7]. Adaptations tested in antenatal populations include mindfulness‐based cognitive therapy [12], the addition of peer support [13], and partner participation [14]. Complementary and alternative medicine (CAM) modalities—such as yoga—are also used in pregnancy, with many women reporting psychological comfort and perceived safety [15]. In Muslim populations, integrating Islamic values and spirituality into care may be helpful; for example, listening to the Qur’an has been examined as a potential means to improve mental well‐being and promote adaptive thinking [16, 17]. More recently, randomized and implementation studies have expanded delivery options to include brief clinic‐delivered IPT and a range of technology‐supported mind–body or mindfulness formats—supervised e‐mindfulness, smartphone‐based programs, and even immersive virtual‐reality sessions—alongside group psychological‐skills models embedded in routine antenatal care, e.g., [18–23].

This narrative review synthesizes evidence on the implementation feasibility and transferability of nonpharmacological interventions for antenatal depression in low‐ and middle‐income countries (LMICs), including Palestine. Recent systematic reviews and meta‐analyses have synthesized nonpharmacological interventions for antenatal or perinatal depression, including a 2025 network meta‐analysis focused on the prevention of antenatal depression [24] and a 2025 meta‐analysis focused on adolescent mothers with perinatal depression and anxiety [25]. However, those syntheses primarily emphasized pooled efficacy and comparative effects and did not primarily address implementation feasibility, readiness for scale‐up in low‐resource antenatal systems, task sharing across midwives, nurses, and primary care workers, culturally or spiritually integrated modalities, or Palestine‐specific service constraints. The present narrative review was therefore designed to complement, rather than replicate, prior evidence syntheses by interpreting the literature through an implementation and transferability lens.

### 1.1. Overview of Antenatal Depression in Palestine

Available Palestinian data suggest a high burden of depressive symptoms in pregnancy, although differences in instruments, thresholds, sampling, and validation limit direct comparison between studies. In the West Bank cross‐sectional study of 327 pregnant women attending antenatal clinics in nine refugee camps, 59.5% were classified as having some degree of depressive symptoms using the patient health questionnaire‐9 (PHQ‐9), including 34.0% with mild symptoms, 17.2% with moderate symptoms, 6.1% with moderately severe symptoms, and 2.1% with severe symptoms [26]. The study did not clearly report validation of the PHQ‐9 specifically among Palestinian pregnant women; therefore, this estimate should be interpreted as a screening‐based symptom estimate rather than a diagnostic prevalence. In Gaza, a cross‐sectional study of 400 pregnant women attending governmental and United Nations Relief and Works Agency primary health care clinics used the 13‐item Beck Depression Inventory. The study reported using an Arabic version previously applied in Palestinian populations and demonstrated internal consistency of 0.89 in the study sample. Depressive symptom levels were classified as mild in 23.3%, moderate in 33.3%, and severe in 18.5% of participants [27]. These estimates should not be interpreted as directly comparable prevalence figures because the studies used different screening instruments, severity classifications, and clinic‐based samples. Multiple contextual risk factors have been described, including patriarchal norms and gender bias (e.g., son preference), unplanned pregnancy, fetal anomalies, labor pain without a support person, exposure to intimate partner violence, higher gravidity and parity, refugee camp residence, low income, and lower educational attainment [26–28]. Health system indicators also point to rising clinical complexity: the proportion of high‐risk pregnancies reportedly increased from 17.5% (2018) to 19.5% (2019) among all Palestinian pregnant women [29].

Intimate partner violence, which is one form of gender‐based violence (GBV), was included above as an individual‐level correlate reported in antenatal studies. At the health system level, the Palestinian annual health report documented a 117% increase in administratively reported GBV cases in 2019 compared with 2017. Many recorded cases involved married women, and husbands were identified as the alleged perpetrators in a substantial proportion of reports [29]. These figures represent recorded or service‐reported cases rather than findings from a population‐based prevalence survey and may therefore have been influenced by service access, reporting practices, and changes in case documentation. In the West Bank antenatal clinic study, exposure to partner violence was examined in relation to depressive symptom severity; however, most reported associations were not statistically significant, and the cross‐sectional design precluded causal inference [26]. The annual report also recorded psychological and combined forms of violence and a small increase in reported mental health conditions from 42.6% in 2018–43.6% in 2019. These separate administrative indicators should not be interpreted as evidence that violence caused the observed increase in mental health reporting. In this context, a political or military conflict should be distinguished from interpersonal violence.

The mental health and psychosocial needs of pregnant women, together with broader social determinants of distress, are compounded by constraints within the Palestinian health system. Despite decades of military occupation, the Israeli Mental Health Law has not been applied in the West Bank and Gaza, and Palestine lacks a dedicated national mental health legal framework [30]. Gaps in continuity and quality of perinatal services have been documented alongside restricted access due to political instability [31]. Mental health services are sparse—provided through only 16 specialized mental and community clinics—and are limited by constrained human resources and insufficient dedicated funding [29, 32, 33]. The capacity for task sharing is constrained by workforce shortages, competing clinical responsibilities, service fragmentation, and limited financing. Recent Palestinian and humanitarian health reports describe substantial disruption of primary, maternal, and mental health services, reinforcing the need for interventions that use existing antenatal contacts without overburdening frontline personnel [34, 35]. Since October 2023, damage to health facilities, population displacement, restrictions on access, shortages of essential resources, and reduced functionality of maternity and primary care services have further narrowed the conditions under which antenatal mental health interventions can be delivered safely and continuously [34]. Regional comparative data indicate that antenatal depression among Arab women in Northern Israel was somewhat higher than among Jewish Israeli women in the same area, underscoring the role of social determinants and minority stressors [36].

Community‐based resources may offer additional entry points for perinatal mental health support. Governmental primary care and United Nations Relief and Works Agency clinics already provide antenatal contacts, while nongovernmental organizations, community workers, social services, family networks, and faith‐based organizations may contribute psychosocial support, referral, outreach, or culturally acceptable engagement. However, the Palestinian sources identified in this review did not provide sufficient information to determine how consistently these resources are integrated into formal antenatal mental health pathways. Mapping their current roles, referral relationships, supervision, and capacity should therefore precede implementation.

#### 1.1.1. Scope and Approach

Given the heterogeneity of nonpharmacological modalities, study designs, and outcome measures across the antenatal depression literature, we undertook an implementation‐oriented narrative review rather than a pooled comparative synthesis. Our purpose was not to determine a single best intervention but to identify intervention formats with potential clinical and implementation relevance and assess their contextual adaptability for antenatal services in resource‐constrained settings. In line with the contemporary trial landscape, we consider both conventional face‐to‐face psychotherapies and technology‐supported delivery formats while interpreting them according to feasibility, supervision needs, cultural fit, and readiness for scale‐up.

#### 1.1.2. Article Structure

We briefly describe our targeted search and selection approach, then present a thematic synthesis by intervention category (psychotherapies; mindfulness/mind–body; culturally/spiritually integrated; acupuncture/adjuncts; and midwife/nurse‐supported counseling), and conclude with implications for practice, policy, and research.

#### 1.1.3. Aim

Narratively synthesize evidence on the effectiveness and implementation feasibility of nonpharmacological interventions for antenatal depression in low‐resource settings, with particular attention to task sharing, cultural adaptation, and applicability to Palestine.

## 2. Methods

### 2.1. Review Design and Reporting Approach

This is a narrative review synthesizing evidence on nonpharmacological interventions for antenatal depression with attention to feasibility in LMICs, including Palestine. Planning and reporting were informed by the scale for the assessment of narrative review articles (SANRA), including clarity of aims, transparent description of the targeted search, explicit selection logic, scientific reasoning, and appropriate presentation [37].

### 2.2. How This Review Differs From Prior Evidence Syntheses

This review differs from prior systematic reviews and meta‐analyses in three ways. First, it focuses on implementation feasibility, transferability, and service fit rather than pooled comparative efficacy. Second, it includes culturally and spiritually integrated approaches, task‐shared delivery models, and supervised digital formats as implementation‐relevant categories for LMIC antenatal care. Third, it interprets the evidence through a Palestine‐oriented health system lens, including workforce constraints, supervision requirements, privacy and referral needs, and readiness for scale‐up in routine antenatal services.

A recent scoping review of strategies for spreading, scaling, and sustaining perinatal mental health interventions in LMICs further identified workforce diversification, integration into routine health services, contextual adaptation, training, supervision, tool development, and stakeholder engagement as central implementation strategies. It also highlighted persistent gaps in evidence on long‐term sustainability and movement beyond early implementation phases [38]. The present review complements that systems‐level synthesis by examining how these considerations apply to specific nonpharmacological interventions for antenatal depression and to the Palestinian context.

### 2.3. Information Sources

Electronic databases were scanned broadly and then refined: PubMed/MEDLINE, ScienceDirect, SpringerLink, JMIR Publications, and Google Scholar (for forward citation chasing and the identification of gray literature). To capture Arabic‐language scholarship, the An‐Najah University Journal for Research was hand‐searched. Reference lists of included papers and field‐relevant reviews were screened for additional studies. Google Scholar screening was limited to the first 200 results sorted by relevance with manual deduplication; no automation tools were used for study selection or data extraction. The search covered the literature up to September 30, 2025.

### 2.4. Search Strategy

Search blocks combined population, condition, and intervention concepts. Representative English terms (adapted per platform syntax) included: (*pregnan* OR antenatal OR “antenatal care”) ^∗^ AND (*depress* OR “depressive symptoms”) ^∗^ AND (*non-pharmacologic* OR psychotherapy OR CBT OR “interpersonal therapy” OR mindfulness OR exercise OR “social support” OR acupuncture OR “complementary and alternative medicine” OR intervention OR prevention OR treatment) ^∗^. Context‐specific strings were also run to assess regional relevance and transferability (e.g., Palestine, “Arab world”, and “Islamic world”), and Arabic terms were used in the An‐Najah search and Google Scholar to surface locally indexed work. All strings were pilot‐tested for recall and precision before full runs. Deduplication was performed in EndNote (v21), followed by manual verification. Full exemplar search strings for each database are provided in Supporting Information 1: File S1.

### 2.5. Eligibility Criteria

Studies were included when they focused on pregnant women in the antenatal period who had a depressive disorder, clinically relevant depressive symptoms established by diagnostic criteria or validated symptom thresholds, or were enrolled in prevention‐oriented studies in which depressive symptoms were a prespecified outcome; evaluated nonpharmacological interventions explicitly targeting depressive symptoms or relevant psychological risk and protective factors, including psychotherapies such as CBT/IPT and midwife‐ or nurse‐delivered brief therapies, mind–body or mindfulness programs, exercise or physical‐activity interventions, social, partner, or community‐support approaches, culturally or spiritually integrated modalities, and adjunctive nondrug strategies such as acupuncture; and used randomized controlled, quasi‐randomized, quasi‐experimental, or prospective single‐group pre–post designs capable of contributing evidence on intervention effects, preliminary effectiveness signals, or implementation feasibility. Randomized and controlled studies were used to support comparative interpretations, whereas uncontrolled single‐group pre–post studies were interpreted only as evidence of within‐group change and preliminary feasibility and were not considered sufficient to establish efficacy (primary outcome: change in depressive symptoms during pregnancy; secondary outcomes: adherence, feasibility, and acceptability). Eligible languages were English and Arabic.

Studies were excluded when they assessed pharmacological or hormonal treatments (e.g., antidepressants and estrogen therapy); examined interventions that did not explicitly target depressive symptoms; enrolled postpartum‐only samples; or relied on highly specialized modalities that demand expertise or infrastructure unlikely to be scalable in LMIC settings (e.g., bright‐light therapy), given the review’s implementation focus on strategies feasible in countries such as Palestine. Publications without extractable depression outcomes were excluded unless they reported prespecified pregnancy, neonatal, or implementation outcomes arising from an otherwise eligible antenatal nonpharmacological intervention program.

### 2.6. Study Selection

Titles and abstracts were screened for relevance, followed by full‐text assessment against the above criteria. Two reviewers independently screened the titles/abstracts and full texts; disagreements were resolved by consensus or a third reviewer. Reference lists of eligible studies and relevant reviews were scanned to identify additional records. Data extraction was performed in duplicate using a piloted form capturing study design, setting, sample, intervention dose, delivery agent, comparator, outcome measures/time points, attrition, and feasibility signals. Inter‐rater agreement was monitored during a calibration phase; persistent discrepancies were adjudicated by consensus.

### 2.7. Data Extraction and Synthesis

From each included study, we charted the country and setting, study design, sample size and participant characteristics, intervention description and delivery provider or platform, comparator, outcome measures and time points, and the direction and consistency of clinical findings. Contextual information was also extracted, where reported, including intervention dose, supervision requirements, integration into routine services, and other delivery features relevant to resource‐constrained settings. A consolidated summary of these characteristics is presented in Supporting Information 2: Table S1.

Given the heterogeneity of the included studies, findings were integrated using a thematic narrative synthesis organized by intervention category: psychotherapies, mindfulness and mind–body interventions, culturally or spiritually integrated interventions, midwife‐ or nurse‐supported counseling, and acupuncture or other adjuncts. Clinical findings were interpreted according to the study design and comparator. Causal or comparative language was reserved for randomized or controlled studies, while findings from uncontrolled pre–post studies were described as preliminary within‐group changes. Implementation considerations were interpreted with reference to Proctor’s implementation outcomes, including acceptability, appropriateness, adoption, feasibility, fidelity, implementation cost, reach, and sustainability [39]. Because these outcomes were not consistently or formally measured across the included studies, the framework was used as an interpretive guide rather than as a formal scoring or study classification system.

No pooled or summary effect estimate was calculated. Guided and unguided digital formats were compared descriptively according to their reported outcomes, adherence, supervision, and service requirements, and no formal weighting score was assigned.

### 2.8. Implementation‐Focused Interpretation

Implementation feasibility and scalability were interpreted cautiously using the contextual and implementation information reported in the primary studies. Particular attention was given to task sharing, integration into routine antenatal services, provider and supervision requirements, intervention dose, digital or material resources, referral arrangements, and reported indicators such as participation, retention, attendance, adherence, or completion. These indicators were treated as preliminary evidence relevant to feasibility rather than as formal measures of acceptability, adoption, or scalability.

Potential scalability was considered in relation to workforce capacity, service integration, supervision, cultural adaptation, infrastructure, reach, cost, and sustainability. Where these outcomes were not reported, no assumptions were made. The review therefore distinguishes small‐scale or service‐level feasibility from demonstrated organizational adoption, population‐level reach, or long‐term sustainability.

### 2.9. Credibility Considerations

As a narrative review, no single universal risk‐of‐bias tool was applied across all included study designs. To support scientific reasoning, peer‐reviewed trials were prioritized, and design limitations (e.g., small samples, nonblinding in psychosocial trials, and reliance on self‐report) were considered when interpreting effects. Gray literature (e.g., theses) and nonpeer‐reviewed sources, when encountered, were used only to contextualize service gaps or implementation issues and were not treated as primary efficacy evidence. For major randomized trials, we conducted a focused, narrative risk‐of‐bias appraisal referencing Cochrane RoB 2 domains (randomization process, deviations from intended interventions, missing outcome data, measurement of outcomes, and selection of reported results). The appraisal was used to qualify the language of interpretation, particularly when distinguishing randomized comparisons from uncontrolled within‐group changes. No numerical risk‐of‐bias score, certainty grade, or formal study weight was assigned.

### 2.10. Study Flow and Counts

**Figure 1:**
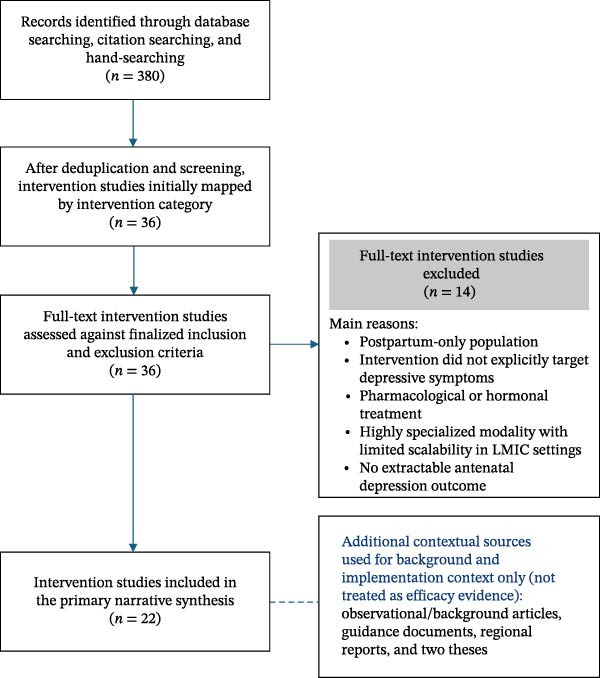
Study‐selection flow diagram. *Note*: The manuscript reports 380 identified records, 36 intervention studies initially mapped after screening, and 22 studies included in the primary narrative synthesis. Intermediate record‐level counts before the 36 mapped intervention studies and reason‐specific exclusion counts were not separately reported.

The search identified 380 records. After deduplication and screening, 36 intervention studies were initially mapped by intervention category to understand the landscape. Applying the finalized inclusion/exclusion criteria focused on antenatal depressive symptoms and feasible nonpharmacological interventions, 22 studies were retained for the primary narrative synthesis (Figure 1). Additional contextual sources (observational/background articles, guidance documents, regional reports, and two theses) informed background and implementation considerations but were not treated as efficacy evidence.

### 2.11. Protocol Registration and Review Updates

No protocol was registered for this narrative review. The final search was completed on September 30, 2025, and no subsequent or living search updates were conducted.

## 3. Results

Twenty‐two intervention studies met the inclusion criteria. Findings are organized by intervention category and describe both clinical outcomes and contextual implementation features, including country and service setting, delivery provider or platform, intervention dose, comparator, supervision, and integration into routine care, where reported. Detailed study characteristics are presented in Supporting Information 2: Table S1, while Supporting Information 3: Table S2 summarizes clinical findings, delivery characteristics, feasibility indicators, implementation gaps, and relevance to Palestine and similar settings.

### 3.1. Acupuncture and Antenatal Depressive Symptoms

Two randomized trials evaluated depression‐specific acupuncture against active comparators. In a pilot RCT of 61 pregnant women diagnosed with major depressive disorder, depression‐specific acupuncture achieved a significantly higher acute‐phase response rate (69%) than massage (32%), while the response rate in the control‐acupuncture group was intermediate at 47%; reductions in Beck Depression Inventory scores from baseline to 1 month were greater with depression‐specific acupuncture than with massage, and responders across arms maintained lower depression scores at 10‐weeks postpartum than nonresponders [40]. In a larger RCT of 150 women receiving 12 sessions over 8 weeks, depression‐specific acupuncture produced greater reductions in symptom severity than control acupuncture or massage; the two control conditions did not differ significantly, with response rates of 37.5% for control acupuncture and 50.0% for massage, supporting the specificity of the point prescription [41].

### 3.2. CBT, IPT, and Related Psychotherapies

Across eight studies, CBT/IPT‐based interventions generally produced favorable findings, although the strength of inference differed between controlled and uncontrolled designs. A U.S. RCT (*N* = 146) showed that a 6‐week, clinic‐integrated CBT program reduced depressive symptoms compared with treatment as usual, with benefits evident among high‐risk African American participants and the broader low‐to‐moderate‐risk cohort [42]. A U.K. pilot RCT (*N* = 36) found that up to 12 sessions of individual CBT added to usual care, initiated at 8–18 gestational weeks, were feasible and improved antenatal depression relative to usual care (Burns et al. [43]). In primigravida women (*N* = 68), eight weekly 1‐h sessions of group CBT produced significant improvements immediately post‐treatment and at 4‐ and 8‐week follow‐ups compared with routine care [44]. In rural Pakistan, a cluster‐randomized trial evaluated a CBT‐based intervention delivered by trained primary health workers within routine maternal and child health services. The intervention reduced antenatal depressive symptoms and improved maternal functioning and selected infant care and health outcomes compared with enhanced routine care, while also demonstrating preliminary feasibility for supervised task sharing in a low‐resource primary care setting [45]. Three IPT studies provided favorable findings, although the strength of inference differed by design. A U.S. controlled clinical trial randomized 50 pregnant outpatients meeting DSM‐IV criteria for MDD to a 16‐week bilingual IPT program or a parenting‐education comparator; 38 participants were included in the analysis. IPT produced significantly greater improvement on the EPDS, BDI, and Hamilton Rating Scale for Depression, and 60% of the IPT group met the study’s recovery criterion [46]; a prospective single‐group pre–post study (*N* = 13) delivering weekly 50‐minute IPT sessions for 16 weeks reported within‐group reductions in depressive symptoms during pregnancy; among the 10 women assessed 3 months postpartum, none reported depressive symptoms. Because the study lacked a control group, the findings provide preliminary evidence of improvement rather than comparative efficacy [47]. In United States obstetrics and gynecology clinics, an evaluator‐blinded randomized controlled trial of 234 pregnant women found that a brief eight‐session IPT intervention produced faster symptom reduction and fewer major depressive disorder diagnoses by term than enhanced usual care, suggesting potential compatibility with clinic‐based delivery [18]. In routine services, a routine‐care pre–post study of internet‐delivered CBT integrated into antenatal services reported within‐group reductions in depressive symptoms across pregnancy. The findings support preliminary service‐level feasibility but do not establish comparative effectiveness because no randomized control group was included [48].

### 3.3. Mindfulness/Mind–Body Programs

A pilot randomized trial of 48 women attending a maternity service compared a mindfulness program with an active pregnancy support program. Only 20 participants completed postprogram measures, and no statistically significant between‐group differences were found. Both groups reported improvements, while qualitative findings and greater practical improvement in present‐moment awareness in the mindfulness group supported preliminary feasibility and acceptability rather than comparative effectiveness [49]. Subsequent multicenter and digital trials broadened the evidence base. In a multicenter RCT, 5299 pregnant women were screened, 1153 had EPDS scores >9, and 460 were included in the trial of an 8‐week supervised electronic mindfulness‐based intervention delivered between 29 and 36 weeks’ gestation. No significant intervention‐by‐time effects were found for general depressive symptoms or anxiety. However, pregnancy‐ and birth‐related anxiety decreased, mindfulness improved, and the proportion of women at risk for an adverse mental health outcome at 6 weeks postpartum was lower in the intervention group [19]. An RCT of 75 pregnant women with heightened distress compared a mobile‐delivered four‐immeasurable mindfulness intervention with a web‐based perinatal education program. The mindfulness group showed greater reductions in EPDS scores after the intervention, which were sustained to 4–6 weeks postpartum [23]. By contrast, a randomized controlled trial involving 219 women with elevated pregnancy distress compared an 8‐week self‐guided online mindfulness intervention (*n* = 109) with care as usual (*n* = 110). Pregnancy distress decreased in both groups, but no statistically significant between‐group difference was found; low intervention adherence indicated potential engagement limitations for unguided delivery [20]. In a randomized controlled trial of 120 women with twin pregnancies, participants were allocated to a 6‐week therapist‐led online mindfulness group intervention, delivered for 120 min weekly, or to usual perinatal care and health education. The intervention prevented worsening of depressive symptoms from late pregnancy to 1 month postpartum and was associated with a lower incidence of low birth weight [21]. In an RCT of 70 pregnant women with EPDS scores of 9–12, daily 14 min immersive virtual reality (IVR) mindfulness and relaxation sessions delivered for 6 weeks reduced depressive and anxiety symptoms compared with standard care. Adherence was high, with 79% completing at least 30 sessions, and 87% reported being very satisfied [50]. A feasibility trial in Pakistan among expectant mothers with a male child preference evaluated a culturally adapted, modified mindfulness program aligned with Islamic teachings and reported within‐participant reductions in antenatal depressive symptoms and perceived stress from baseline to postintervention and short‐term follow‐up. The study included 84 participants, with 42 assigned to the modified mindfulness intervention and 42 to routine hospital care. Depression and perceived stress scores decreased significantly over time in the intervention group, while no significant changes occurred in the control group. Because the allocation procedure and feasibility outcomes were not reported with the rigor of a definitive randomized effectiveness trial, the findings should be interpreted as preliminary comparative and implementation evidence rather than definitive efficacy [51].

Mindfulness dose and delivery varied substantially across studies. Interventions ranged from brief visit‐aligned or immersive virtual‐reality sessions to structured programs lasting approximately six to 8 weeks. The twin‐pregnancy trial delivered 120 min therapist‐led group sessions weekly for 6 weeks, while the Hulsbosch program was an 8‐week self‐guided online intervention. Other studies used supervised electronic or smartphone delivery with between‐session practice. Because frequency, session duration, home practice, guidance, and adherence were not reported consistently, the review cannot identify an optimal mindfulness dose. These variables are now reported individually in Supporting Information 2: Table S1 and the implementation table.

### 3.4. Midwife/Nurse‐Supported Counseling

In an ultra‐Orthodox Israeli community, a prospective pre–post study evaluated brief nondirective counseling delivered by trained nurses within routine antenatal care. Of 160 women screened using the EPDS, 19 scored ≥10 antenatally, and nurses provided supportive counseling to 40 women. EPDS scores decreased from antenatal assessment to 6 weeks postpartum, with a greater reduction among women whose antenatal EPDS score was ≥10. The findings provide preliminary evidence that brief nurse‐supported counseling can be delivered within routine antenatal care, but the uncontrolled design does not establish comparative efficacy [52].

### 3.5. Culturally and Spiritually Integrated Interventions

Two Iranian randomized publications evaluated Qur’an‐based interventions but reported different outcome domains. Mirghafourvand et al. [53] randomized 168 Muslim pregnant women at 25–28 weeks’ gestation to Qur’an listening with Persian translation, Qur’an listening without translation, or routine pregnancy care. Women with EPDS scores ≥12 were excluded and referred for mental health care. The study focused on pregnancy and neonatal outcomes: preterm delivery and cesarean delivery were numerically less frequent in the intervention groups, but the differences were not statistically significant, and neonatal anthropometric indices did not differ between groups. Jabbari et al. [54] evaluated depressive symptoms, perceived stress, and state and trait anxiety using the EPDS, PSS, and STAI. Both Qur’an‐listening groups had significantly lower scores than the control group at follow‐up, with no significant difference between the two intervention groups. A quasi‐experimental comparison (*N* = 30) reported that both a mindfulness‐based Islamic spiritual program and group CBT reduced antenatal depression compared with control, with the Islamic mindfulness program showing the larger effect [55]. Complementing these culturally consonant modalities, the SM‐ART RCT in Pakistan randomized 200 generally recruited pregnant women to 6 weekly group sessions delivered by trained community midwives or to standard antenatal care. The intervention group reported modestly lower depressive symptom scores and greater resilience after 6 weeks; however, elevated depression was not an eligibility requirement, and only 45 of 100 intervention participants attended all six sessions [22].

### 3.6. Implementation Feasibility and Scalability Across Intervention Categories

Implementation outcomes were reported inconsistently across the included studies. Preliminary feasibility was suggested by brief clinic‐based delivery, integration into antenatal or obstetric services, task sharing with primary health workers or nurses, group‐based formats, and selected supervised digital interventions. Reported indicators included participation, retention, attendance, adherence, intervention completion, compatibility with routine antenatal contacts, and the use of existing clinical or community‐based personnel. However, these indicators were generally reported descriptively and were not equivalent to formal assessments of implementation outcomes. Most studies focused primarily on depressive symptoms and did not formally assess acceptability, fidelity, implementation cost, organizational adoption, population reach, or long‐term sustainability. Culturally adapted and spiritually integrated interventions suggested potential contextual relevance, but cultural acceptability, appropriateness, and informed patient choice were rarely measured directly. Provider training, supervision requirements, digital access, referral capacity, and availability of protected staff time also varied across intervention categories and were often incompletely reported. Overall, the evidence supports cautious judgments about small‐scale or service‐level feasibility but does not establish system‐wide scalability, sustained organizational adoption, or long‐term sustainability.

### 3.7. Cross‐Category Synthesis

Across intervention categories, the direction of findings generally favored nonpharmacological care, although the strength of inference varied substantially by study design, comparator, sample size, and completeness of implementation reporting. A thematic synthesis of effects, delivery, and implementation considerations by intervention category is shown in Supporting Information 3: Table S2. The most robust comparative signals were derived from randomized trials of CBT/IPT and depression‐specific acupuncture [18, 40, 41, 45], complemented by mixed evidence from supervised digital mindfulness and structured group formats, including favorable findings in selected therapist‐led and mobile‐delivered trials but no general depression effect in the Hassdenteufel eMBI trial [19, 21, 50]. By contrast, the unguided online mindfulness trial showed no significant between‐group advantage and was limited by low adherence [20]. Evidence for nurse‐supported counseling suggests preliminary service delivery feasibility in routine antenatal settings, although the uncontrolled design does not establish comparative efficacy [52]. Overall, the contribution of this review lies in identifying which formats appear most amenable to task sharing, cultural adaptation, and staged implementation in resource‐constrained antenatal systems rather than in establishing a pooled hierarchy of efficacy.

## 4. Discussion

This narrative review synthesized evidence from 22 intervention studies evaluating nonpharmacological strategies for antenatal depression through an implementation‐oriented lens relevant to resource‐constrained settings. Rather than ranking interventions by pooled comparative efficacy, the review identified intervention formats with potential clinical and service relevance for LMIC antenatal systems, including Palestine. Overall, the most consistent comparative evidence supported CBT/IPT and depression‐specific acupuncture, while selected supervised mindfulness formats, midwife‐/nurse‐supported counseling, culturally and spiritually integrated approaches, and task‐shared group programs showed additional promise. However, implementation evidence was concentrated mainly on early indicators such as attendance, adherence, completion, task sharing, and integration into routine antenatal contacts. Formal evidence regarding acceptability, fidelity, organizational adoption, population reach, implementation cost, and sustainability remained limited. The findings therefore support preliminary judgments about service‐level deliverability rather than firm conclusions about system‐wide scalability.

This distinction is particularly important for Palestine, where service fragmentation, constrained mental health infrastructure, and restricted access shape what can realistically be incorporated into antenatal care pathways [30, 31]. Implementation planning is also limited by the routine availability of standardized antenatal mental health indicators, which affects estimation of baseline need, equity monitoring, workforce planning, cost analysis, and assessment of population reach. Strengthening screening, referral, and outcome‐monitoring systems should therefore accompany future implementation rather than follow it.

Digital interventions may extend access and reduce some demands on specialist personnel, but they should not be treated as inherently scalable. Their implementation depends on equitable access to suitable devices, reliable connectivity or offline functionality, digital literacy, privacy during use, data protection, technical support, adherence strategies, and clear procedures for responding to clinical deterioration. These conditions were not consistently evaluated in the included studies. In Palestine and other settings affected by humanitarian disruption, intermittent electricity, unstable connectivity, displacement, and limited private space may further compromise continuity and safe use. These considerations align with the scale‐up strategies identified by Sanfilippo et al. [38], including workforce diversification, integration into maternal and primary health services, contextual adaptation, structured training and supervision, and stakeholder engagement. However, the limited reporting of cost, system‐wide penetration, long‐term financing, workforce retention, and sustainability means that symptom improvement or small‐scale feasibility should not be interpreted as evidence that an intervention can be spread or maintained at scale.

Psychological therapies represented the strongest and most implementation‐relevant evidence category. Across individual, group, clinic‐integrated, task‐shared, brief, and internet‐delivered formats, CBT/IPT generally produced favorable findings, although the strength of inference differed by design [18, 42–48]. The randomized evidence supports comparative benefit, while the uncontrolled Spinelli and Mahoney studies provide only preliminary evidence of within‐group change and service‐level feasibility. From an implementation perspective, the principal value of this evidence is the flexibility of CBT/IPT delivery: brief formats may fit obstetric schedules, group approaches may reduce provider burden, and supervised task sharing may extend access where specialist capacity is limited. The rural Pakistan trial is especially relevant because it demonstrated that a CBT‐based intervention could be delivered by trained primary health workers within routine maternal and child health services, while the brief IPT trial indicated compatibility with busy obstetric clinics [18, 45].

The acupuncture evidence was narrower but comparatively robust. Two randomized trials supported depression‐specific acupuncture over active comparators, suggesting that a time‐limited and protocolized intervention may be clinically useful where trained practitioners, safety procedures, and governance structures are available [40, 41]. Its relevance to low‐resource systems is therefore conditional: the intervention may be feasible in selected settings but is unlikely to be broadly transferable where practitioner supply, supervision, or regulatory oversight is limited.

Mindfulness and mind–body interventions showed greater variation in format, intensity, guidance, and outcome. Findings varied across clinic‐embedded, supervised electronic, mobile‐delivered, therapist‐led group, and IVR formats [19, 21, 23, 49, 50]. The Hassdenteufel eMBI trial did not demonstrate a significant effect on general depressive symptoms or anxiety, although pregnancy‐ and birth‐related anxiety decreased and mindfulness improved. By contrast, the smaller mobile‐delivered Leng trial reported sustained reductions in EPDS scores compared with an active web‐based perinatal education control. The unguided Hulsbosch et al. [20] intervention did not demonstrate a between‐group advantage and was limited by low adherence. The culturally adapted feasibility study by Badil et al. [51] reported greater longitudinal improvement in the intervention group than in a concurrent routine‐care control group; however, the study design and reporting do not support definitive conclusions regarding efficacy or scalability. Taken together, these studies suggest that supervision, therapist contact, structured delivery, and adherence support may be more important than the digital platform itself. However, because intervention frequency, session duration, home practice, guidance, and engagement were not compared directly, no optimal mindfulness dose or delivery intensity can be established.

The evidence for midwife‐/nurse‐supported counseling and culturally integrated care is particularly relevant to task sharing, but it should be interpreted cautiously. The service‐embedded nursing model described by Glasser et a. [52] provides preliminary evidence that brief supportive counseling can be delivered within routine antenatal care, although the uncontrolled design does not establish comparative efficacy. Qur’an listening with or without translation was associated with lower depressive symptom, perceived stress, and anxiety scores than routine care in a randomized study of Muslim pregnant women with EPDS scores below 12 [54]. A separate publication examining pregnancy outcomes found no statistically significant effects on preterm delivery, cesarean delivery, or neonatal anthropometric indices [53]. Islamic‐spiritual mindfulness and the SM‐ART group intervention also reported favorable findings, although the populations, study designs, and strength of inference varied [22, 55]. These approaches may have contextual relevance for some Muslim women, but their cultural or religious acceptability should not be assumed. Future implementation should assess acceptability, appropriateness, engagement, informed choice, and fidelity using validated implementation measures, participation and completion indicators, qualitative feedback, and adverse event monitoring. Equivalent nonfaith‐based options should also remain available.

The observed findings may be understood through several shared mechanisms, although these mechanisms were not consistently measured. CBT and IPT may reduce distress through cognitive restructuring, behavioral activation, problem‐solving, interpersonal support, and improved social functioning. Mindfulness may strengthen attentional regulation and reduce stress reactivity, while supportive counseling may normalize distress and mobilize coping through a structured therapeutic relationship. The included acupuncture trials established clinical symptom differences but did not determine the biological mechanisms responsible for those changes. Likewise, culturally and religiously integrated interventions may improve perceived relevance, meaning, or engagement, but these mechanisms require prospective assessment rather than assumption. The cluster trial reporting maternal and infant benefits also suggests that effective antenatal depression care may generate broader family and early life benefits [45]. Qualitative evidence emphasizing the peer connection and perceived support during pregnancy further supports the inclusion of social engagement within psychological interventions [56].

Translation to Palestine therefore requires a staged and locally evaluated service model rather than the direct adoption of any single intervention. Observational evidence linking family and partner support with lower depressive symptoms highlights the relevance of the social context, but these associations should not be interpreted as treatment effects [57–59]. A practical pathway would combine routine symptom screening, confidential assessment of self‐harm and violence risk, clear referral procedures, and stepped access to care. Brief group CBT, visit‐aligned mindfulness, or culturally consonant programs may be evaluated as initial low‐intensity options, with individual CBT or IPT available for persistent or more severe symptoms, and acupuncture reserved for settings with appropriate expertise and governance [40–44, 46, 49, 54, 55]. Supervised e‐mindfulness, smartphone‐based programs, brief IPT, IVR, and SM‐ART may offer additional delivery options where infrastructure, privacy, supervision, and adherence support are sufficient [18–23, 50, 51]. Across these options, task sharing to midwives, nurses, and primary health care workers should be accompanied by competency‐based training, ongoing supervision, fidelity support, and functioning referral links rather than viewed simply as redistribution of work [45, 52].

### 4.1. Implications and Recommendations for Palestine and Similar Settings

The findings support a staged, locally evaluated approach rather than immediate system‐wide implementation or a single intervention model. Antenatal services should first establish confidential symptom screening using a validated instrument such as the EPDS, assessment of self‐harm and violence risk, clear referral pathways, and access to supervised care. Brief CBT‐ or IPT‐based approaches delivered individually or in groups appear to be the most suitable initial candidates for task‐shared implementation, provided that nurses, midwives, primary care workers, or community providers receive competency‐based training, ongoing supervision, and simple fidelity support.

For women with mild to moderate symptoms, services may initially evaluate brief group CBT, visit‐aligned mindfulness, or culturally and spiritually integrated programs. These approaches should be offered according to informed patient preference and assessed for acceptability, appropriateness, engagement, and clinical benefit rather than assumed to be universally suitable. Women with persistent or more severe symptoms should be referred or stepped up to individual CBT or IPT delivered by appropriately trained providers under specialist supervision. Protocolized depression‐specific acupuncture should remain an optional adjunct where trained practitioners, safety procedures, and clinical governance are available. Equivalent nonfaith‐based options should remain available to preserve meaningful choice and avoid assumptions based on religious or cultural identity.

Digital options, including supervised e‐mindfulness, smartphone‐delivered programs, and brief IVR sessions, may extend access but should be introduced only after assessing device availability, connectivity or offline functionality, digital literacy, privacy, data protection, technical support, and clinical escalation procedures. Programs should also consider reminders, brief check‐ins, and practical support for data access where these are required to maintain engagement.

Implementation should be supported through task sharing, short, competency‐based training, ongoing supervision, and integration into routine antenatal contacts. Safety procedures should include confidential enquiry and safety planning for intimate partner violence, urgent referral when required, and coordination with specialist, social welfare, and community services. Culturally or spiritually integrated interventions should be codesigned with pregnant women and relevant clinical, community, and faith‐based stakeholders while preserving voluntariness and informed choice.

Initial Palestinian pilots should measure depressive symptoms alongside implementation outcomes. At minimum, evaluations should document recruitment and population reach, attendance and completion, patient and provider acceptability, cultural appropriateness, service adoption, training and supervision time, fidelity, referral uptake, adverse events, delivery cost, and maintenance after external support ends. Mixed‐methods research involving pregnant women, nurses, midwives, community organizations, humanitarian providers, and policymakers is needed to determine how interventions should be adapted and whether they remain feasible during service disruption, displacement, and conflict.

## 5. Limitations

This review focused on nonpharmacological interventions for antenatal depression that are realistically implementable in resource‐constrained settings similar to Palestine. Several factors limit the certainty and generalizability. First, although multiple randomized trials were available, several studies were small or pilot‐scale, and some used prospective pre–post designs without control groups, which reduces causal confidence for those specific findings. Second, the review included heterogeneous intervention categories, provider backgrounds, delivery formats, outcome measures, and study designs. This diversity was expected and appropriate for the broad narrative aim and was not treated as a limitation arising from an inability to pool estimates. However, it constrained direct comparisons across interventions and made judgments regarding relative implementation readiness dependent on the completeness and quality of reporting. Third, follow‐up beyond the antenatal period was uncommon, so the durability of benefits into the postpartum months remains unclear. Fourth, while evidence from LMICs strengthens external validity for resource‐constrained services, none of the efficacy trials were conducted in Palestine, and relatively few originate from the broader Arab region; consequently, transferability still relies on contextual judgment and cultural adaptation. Fifth, implementation outcomes were reported inconsistently across the included studies, particularly fidelity, cost, organizational adoption, population reach, and sustainability. Proctor’s implementation outcomes framework was used as an interpretive guide and could organize only the information reported in the primary studies; it could not compensate for implementation outcomes that had not been measured. Sixth, mindfulness interventions varied in frequency, session duration, home practice expectations, therapist involvement, and digital support. Although such variation is appropriate to examine within a narrative review, inconsistent reporting and the absence of direct comparisons between different intervention doses prevented conclusions about which components or levels of intensity may be most suitable for particular service contexts. Seventh, studies of culturally or spiritually integrated interventions generally assessed depressive symptoms more consistently than acceptability, appropriateness, informed choice, or intervention fidelity. Therefore, conclusions regarding cultural or religious fit remain provisional and should not be generalized to all women within the same cultural or faith community. Finally, because no included intervention study was conducted in Palestine and few were undertaken in active humanitarian settings, the applicability of these interventions during severe service disruption, displacement, or conflict requires local codesign and pragmatic evaluation before broader implementation.

## 6. Conclusion

Several nonpharmacological interventions reduced antenatal depressive symptoms in controlled studies, while other formats provided preliminary or mixed findings; implementation readiness nevertheless remains uncertain. CBT and IPT have the strongest combination of comparative evidence and potential for task‐shared delivery. Mindfulness, nurse‐supported counseling, culturally or spiritually integrated interventions, acupuncture, and digital formats may provide additional options when matched to patient preferences, provider capacity, infrastructure, and governance. Evidence regarding implementation cost, system‐wide reach, fidelity at scale, and sustainability remains limited. For Palestine and similar humanitarian or resource‐constrained settings, the most defensible next step is not immediate broad rollout but locally codesigned, supervised, and pragmatically evaluated integration within antenatal services.

**Nomenclature**
EPDS: Edinburgh postnatal depression scale
BDI: Beck Depression Inventory
CBT: Cognitive behavioral therapy
IPT: Interpersonal psychotherapy
RCT: Randomized controlled trial
OB/GYN: Obstetrics and gynecology
SANRA: Scale for the assessment of narrative review articles
RoB 2: Cochrane risk of bias 2 tool
LMICs: Low‐ and middle‐income countries
PHIC: Palestinian Health Information Center
WHO: World Health Organization
CAM: Complementary and alternative medicine
eMBI: electronic mindfulness‐based intervention
IVR: Immersive virtual reality
SM‐ART: Safe motherhood—accessible resilience training
GBV: Gender‐based violence
DSM‐IV: Diagnostic and statistical manual of mental disorders, fourth edition
PPD: Postpartum depression
TAU: Treatment as usual
MDD: Major depressive disorder
PHQ‐9: Patient health questionnaire‐9.

## Author Contributions

**Sawsan Saeed Abd Al Rahman Shqair:** conceptualization, methodology/search strategy design, database searches and record management, screening (titles/abstracts and full texts), data extraction and charting, synthesis and interpretation, writing – original draft. **Mohammed Hayek:** conceptualization, methodology/search strategy design, verification of a random 20% sample, synthesis and interpretation, writing – original draft, supervision. **Loai M. Zabin:** methodology/search strategy design, database searches and record management, data extraction and charting, data extraction and charting, writing – review and editing. **Shurouq Ghalib Qadous:** database searches and record management, screening (titles/abstracts and full texts), writing – review and editing. **Mohammad Marie:** eligibility adjudication for discrepant cases, synthesis and interpretation, writing – review and editing, supervision. **Jamal Qaddumi:** eligibility adjudication for discrepant cases, writing – review and editing. **Adnan L. Sarhan:** writing – review and editing.

## Funding

This research received no specific grant from any funding agency in the public, commercial, or not‐for‐profit sectors.

## Disclosure

All authors read and approved the final manuscript and agree to be accountable for all aspects of the work. All scientific content, interpretation, and conclusions were developed, reviewed, and verified by the authors.

## Ethics Statement

This manuscript is a narrative review of published literature and did not involve any new studies with human participants or animals. Therefore, ethics committee approval and informed consent were not required. The Declaration of Helsinki does not apply to this review itself; however, where reported, the included primary studies stated that they had received ethics approval and were conducted in accordance with the Declaration of Helsinki.

## Conflicts of Interest

The authors declare no conflicts of interest.

## Supporting Information

Additional supporting information can be found online in the Supporting Information section.

## Supporting information


**Supporting Information 1** File S1 provides the detailed search strategies used for the selected databases and other information sources.

**Supporting Information 2** Table S1 presents the characteristics of the included intervention studies, including study design, sample, assessment criteria, intervention dose, comparator, outcomes, and principal findings.

**Supporting Information 3** Table S2 summarizes the clinical effects, delivery characteristics, feasibility indicators, implementation gaps, and relevance of each intervention category to Palestine and similar resource‐constrained settings.

## Data Availability

No new datasets were generated or analyzed in this study. All data supporting the findings of this study are derived from published sources cited in the article. Full platform‐specific search strategies are provided in Supporting Information 1: File S1.
