# Supplementary material for: A Narrative Review of Implementing Nonpharmacological Interventions for Antenatal Depression in Low Resource Settings With Implications for Palestine
Source: Depress Anxiety. 2026 Aug 11;2026:4692841. doi: 10.1155/da/4692841 (PMC13462660; doi:10.1155/da/4692841)
Supplement: Supplementary file 1 [file DA-2026-4692841-s001.docx]

**Databases, limits, and exemplar search strategies**

**PubMed / MEDLINE**

**Platform/fields:** Boolean with field tags; MeSH where useful
**Last searched:** 30 Sep 2025
**Coverage:** Inception–2025
**Limits applied (at query or screening):** Humans; Female; Pregnancy; English or Arabic; article types not hard-limited in the query (screened at full text for interventional designs)

**Global (non-pharmacological antenatal interventions)**

((pregnan*[tiab] OR antenatal[tiab] OR prenatal[tiab])

AND (depress*[tiab] OR "Depression"[Mesh] OR "Depressive Disorder"[Mesh]) AND (non-pharmacolog*[tiab] OR psychotherapy[tiab] OR "Cognitive Behavioral Therapy"[tiab] OR CBT[tiab] OR "Interpersonal Psychotherapy"[tiab] OR IPT[tiab] OR mindfulness[tiab] OR "mind–body"[tiab] OR exercise[tiab] OR "social support"[tiab] OR acupuncture[tiab] OR "complementary and alternative medicine"[tiab] OR CAM[tiab] OR intervention*[tiab] OR treatment*[tiab])) AND ("Pregnancy"[Mesh] OR pregnan*[tiab] OR antenatal[tiab] OR prenatal[tiab]) AND (random*[tiab] OR trial[tiab] OR "Randomized Controlled Trial"[Publication Type] OR "clinical trial"[tiab] OR "quasi-experimental"[tiab] OR "pre–post"[tiab] OR "before and after"[tiab]) NOT (postpartum[tiab] OR postnatal[tiab])

**Context-specific (Palestine/Arab/Islamic)**

((pregnan*[tiab] OR antenatal[tiab] OR prenatal[tiab]) AND depress*[tiab] AND (psychotherapy[tiab] OR CBT[tiab] OR IPT[tiab] OR mindfulness[tiab] OR "social support"[tiab] OR acupuncture[tiab]) AND (Palestin*[tiab] OR "Arab world"[tiab] OR Islam*[tiab])) AND (random*[tiab] OR trial[tiab] OR "quasi-experimental"[tiab] OR "pre–post"[tiab])

**ScienceDirect (Elsevier)**

**Fields:** Title/Abstract/Keywords
**Last searched:** 30 Sep 2025
**Coverage:** Inception–2025
**Limits:** Article type = Research/Clinical Trials; Language = English or Arabic

**Global**

(TITLE-ABS-KEY(pregnan* OR antenatal OR prenatal) AND TITLE-ABS-KEY(depress*) AND TITLE-ABS-KEY("non-pharmacolog*" OR psychotherapy OR "cognitive behavio*" OR CBT OR "interpersonal therap*" OR IPT OR mindfulness OR "mind-body" OR exercise OR "social support" OR acupuncture OR CAM OR intervention OR treatment OR prevention)) AND TITLE-ABS-KEY(random* OR trial OR "quasi-experimental" OR "pre-post" OR "before and after") AND NOT TITLE-ABS-KEY(postpartum OR postnatal)

**Context**

(TITLE-ABS-KEY(pregnan* OR antenatal OR prenatal) AND TITLE-ABS-KEY(depress*) AND TITLE-ABS-KEY(psychotherapy OR CBT OR IPT OR mindfulness OR "social support" OR acupuncture) AND TITLE-ABS-KEY(Palestin* OR "Arab world" OR “rehabilitation” OR Islam*))

**SpringerLink**

**Search style:** Boolean with “all words”/“exact phrase” fields
**Last searched:** 30 Sep 2025
**Coverage:** Inception–2025
**Limits:** Article/Chapter; disciplines Medicine/Public Health

**Global**

(pregnan* OR antenatal OR prenatal) AND depress* AND (non-pharmacolog* OR psychotherapy OR "cognitive behavioral" OR CBT OR "interpersonal psychotherap*" OR IPT OR mindfulness OR "mind-body" OR exercise OR "social support" OR acupuncture OR CAM OR intervention OR treatment OR prevention) AND (random* OR trial OR quasi-experimental OR pre-post OR "before and after") NOT (postpartum OR postnatal)

**Context**

(pregnan* OR antenatal OR prenatal) AND depress* AND (psychotherapy OR CBT OR IPT OR mindfulness OR "social support" OR acupuncture) AND (Palestin* OR "Arab world" OR “rehabilitation” OR Islam*)

**JMIR Publications (journal site search)**

**Fields:** All fields; Boolean
**Last searched:** 30 Sep 2025
**Coverage:** Inception–2025
**Limits:** Research/Randomized/Quasi-Experimental (protocols screened out at full text)

**Global**

(pregnan* OR antenatal OR prenatal) AND (depress* OR "depressive symptoms") AND (CBT OR "interpersonal" OR IPT OR mindfulness OR "mind-body" OR "social support" OR acupuncture OR CAM OR intervention OR treatment OR prevention) AND (random* OR trial OR quasi OR "pre-post" OR "before and after") NOT (postpartum OR postnatal)

**Context**

(pregnan* OR antenatal OR prenatal) AND depress* AND (CBT OR IPT OR mindfulness OR "social support" OR acupuncture) AND (Palestin* OR Islam* OR "Arab world" OR “rehabilitation”)

**Google Scholar**

**Style:** Boolean (lenient), quoted phrases; used for catch-all discovery and forward citation chasing
**Last searched:** 30 Sep 2025
**Coverage:** 2000–2025 (year filter)
**Limits:** “Since 2000”; exclude patents/citations in UI

**Global (broad net)**

"antenatal" OR prenatal pregnancy depress* non-pharmacologic OR psychotherapy OR CBT OR IPT OR mindfulness OR "social support" OR acupuncture randomized OR trial OR "quasi-experimental" OR "pre-post" -postpartum -postnatal.

**Context/Arabic probe**

"اكتئاب ما قبل الولادة" OR "اكتئاب الحمل" AND ("تدخل غير دوائي" OR علاج نفسي OR يقظة ذهنية OR "دعم اجتماعي" OR "وخز بالإبر") AND (فلسطين OR "العالم العربي" OR "العالم الإسلامي")

**An-Najah University Journal for Research (hand-search, Arabic)**

**Approach:** Arabic keywords; manual scan of titles/abstracts; Health/Medical Sciences section
**Last searched:** 30 Sep 2025
**Coverage:** Inception–2025
**Queries (run separately):**

1. “اكتئاب ما قبل الولادة” AND (“تدخل غير دوائي” OR علاج نفسي OR يقظة ذهنية OR “دعم اجتماعي” OR “وخز بالإبر”)
2. “اكتئاب الحمل” AND (“تدخل غير دوائي” OR علاج نفسي OR يقظة ذهنية OR “دعم اجتماعي” OR “وخز بالإبر”)
3. فلسطين AND (“اكتئاب ما قبل الولادة” OR “اكتئاب الحمل”)

**Operational notes (applied consistently)**

- Truncation/stemming used where supported (e.g., depress*, pregnan*, non-pharmacolog*, cognitive behavio*).
- Antenatal/prenatal/pregnancy terms combined to maximize recall; postpartum-only records excluded at query where possible and at full-text screening otherwise.
- Study-design terms used to surface interventional designs (randomized/controlled; quasi-experimental; prospective pre–post). Final inclusion required confirmation that depressive symptoms were measured during pregnancy.
- Context-specific runs broadened geography terms (Palestine/Arab/Islamic/rehabilitation) but retained the same intervention and design logic.
- Reference lists of included trials and relevant reviews were hand-searched; forward citations of anchor trials (IPT/CBT/acupuncture/mindfulness) were checked via Google Scholar.
- Results were exported (RIS/CSV or spreadsheet), de-duplicated, and screened per the inclusion/exclusion criteria in the Methods.
