# Supplementary material for: A Narrative Review of Implementing Nonpharmacological Interventions for Antenatal Depression in Low Resource Settings With Implications for Palestine
Source: Depress Anxiety. 2026 Aug 11;2026:4692841. doi: 10.1155/da/4692841 (PMC13462660; doi:10.1155/da/4692841)
Supplement: Supplementary file 2 [file DA-2026-4692841-s002.docx]

**Supplementary Table S1.** *Characteristics of the included intervention studies*

| Study (country) | Design | Sample, population, and depression/distress assessment | Gestational timing (when stated) | Intervention (dose, delivery agent) | Comparator | Primary outcome focus | Key finding(s) |
| --- | --- | --- | --- | --- | --- | --- | --- |
| Manber et al., 2004 (USA) | Randomized controlled trial (pilot) | N = 61 pregnant women meeting criteria for major depressive disorder and scoring ≥14 on the 17-item Hamilton Rating Scale for Depression; the Beck Depression Inventory was also used to assess symptom change. | During pregnancy; 8-week acute treatment, with 10-week postpartum follow-up reported for responders. | Depression-specific acupuncture delivered over 8 weeks using a standardized but individually tailored point prescription. | Massage; control acupuncture | Antenatal depressive symptoms, including Beck Depression Inventory change | Higher acute response with depression-specific acupuncture (69%) than massage (32%); the control-acupuncture response was 47%. Beck Depression Inventory reduction was greater than with massage, and responders maintained lower depression scores at 10 weeks postpartum. |
| Manber et al., 2010 (USA) | Randomized controlled trial | N = 150 pregnant women meeting DSM-IV criteria for major depressive disorder; depression severity was assessed using the Hamilton Rating Scale for Depression. | 8-week treatment | Depression-specific acupuncture; 12 sessions over 8 weeks | Control acupuncture; massage | Antenatal depressive symptoms | Depression-specific acupuncture produced a greater rate of symptom reduction than the combined controls and control acupuncture. Response rates were 63.0% for depression-specific acupuncture, 37.5% for control acupuncture, and 50.0% for massage; the two control groups did not differ significantly. |
| Jesse et al., 2015 (USA) | Randomized controlled trial | N = 146 rural, minority, and low-income pregnant women; high risk was defined as EPDS ≥10 and low-to-moderate risk as EPDS 4–9. Depressive symptoms were assessed using the EPDS and BDI-II. | During pregnancy; baseline, post-treatment, and 1-month follow-up. | Culturally tailored group CBT integrated into prenatal clinics; 6-week program. | Treatment as usual | Antenatal depressive symptoms | Both groups improved on the EPDS. No overall treatment effect was found among all high-risk women, but CBT produced greater improvement among high-risk African American women and greater BDI-II improvement in the low-to-moderate-risk group. The clinic-integrated intervention was feasible, although attrition was high. |
| Burns et al., 2013 (UK) | Pilot randomized controlled trial | N = 36 pregnant women meeting ICD-10 criteria for depression; 18 were randomized to CBT plus usual care and 18 to usual care. | Initiated at 8–18 weeks of gestation | Individual cognitive behavioral therapy, up to 12 sessions, plus usual care | Usual care | Antenatal depressive symptoms | Thirteen of 18 women assigned to CBT completed at least nine sessions. At the end of pregnancy, 11/16 women receiving CBT plus usual care and 5/13 receiving usual care no longer met ICD-10 depression criteria; the pilot was not powered for definitive effectiveness conclusions. |
| Mehri et al., 2019 (Iran) | Clinical trial with group allocation | N = 68 primigravida women aged 15–45 years at 22–26 weeks’ gestation with EPDS scores of 10–15; participants were randomly allocated to intervention and control groups, 34 per group. | 22–26 weeks’ gestation; outcomes assessed immediately and at 4 and 8 weeks after the intervention. | Group cognitive behavioral therapy; eight weekly 1-hour sessions | Routine care | Antenatal depressive symptoms | Depressive-symptom scores differed significantly between groups immediately and at 4 and 8 weeks after the intervention, favoring the self-care-based group CBT intervention. |
| Rahman et al., 2008 (Pakistan) | Cluster randomized controlled trial with task sharing | N = 903 married women aged 16–45 years with depression identified during the third trimester in 40 rural union-council clusters; 463 were allocated to intervention clusters and 440 to control clusters. | Third trimester through 12 months postpartum. | CBT-based Thinking Healthy Programme delivered by trained Lady Health Workers: weekly sessions during the final month of pregnancy, three sessions during the first postnatal month, and nine monthly sessions thereafter. | Enhanced routine care delivered through an equal schedule of visits by routinely trained Lady Health Workers. | Antenatal depressive symptoms and maternal and infant outcomes | Maternal depression was substantially lower in the intervention group at 6 and 12 months postpartum, with improvements in maternal functioning and selected infant-care outcomes. No significant between-group differences were found in infant weight or height at 6 or 12 months. |
| Spinelli and Endicott, 2003 (USA) | Randomized controlled clinical trial | N = 50 outpatient pregnant women meeting DSM-IV criteria for major depressive disorder; 38 remained and were included in the analysis. Depression was assessed using the EPDS, BDI, Hamilton Rating Scale for Depression, and Clinical Global Impression. | During pregnancy; 16-week treatment. | Bilingual interpersonal psychotherapy delivered over 16 weeks. | Didactic parenting education program. | Antenatal depressive symptoms and recovery. | IPT produced significantly greater improvement than parenting education on all three mood measures at treatment completion; 60% of the IPT group met recovery criteria based on the Clinical Global Impression. |
| Spinelli, 1997 (USA) | Prospective single-group pre-post study | N = 13 pregnant women meeting Diagnostic and Statistical Manual of Mental Disorders, Third Edition, Revised criteria for major depressive disorder | 16-week program; 3-month postpartum follow-up | Interpersonal psychotherapy; weekly 50-minute sessions for 16 weeks | None | Antenatal depressive symptoms | Within-group reductions in depressive symptoms were reported during pregnancy; among 10 women assessed at 3 months postpartum, none reported depressive symptoms. The uncontrolled design precluded comparative efficacy conclusions. |
| Beattie et al., 2017 (UK) | Pilot randomized trial | N = 48 pregnant women attending a maternity service; depressive symptoms were assessed using the EPDS and perceived stress using the Perceived Stress Scale. No elevated depression or distress threshold was required for eligibility. | During pregnancy; post-treatment assessment | Mindfulness-based program compared with an active pregnancy-support program; | Pregnancy-support program | Feasibility, acceptability, perceived stress, depressive symptoms, present-moment awareness, and birth outcomes. | Only 9 women in the mindfulness group and 11 in the pregnancy-support group completed post-program measures. There were no statistically significant between-group differences. Both groups improved on self-reported stress-related measures, while the mindfulness group showed greater practical improvement in present-moment awareness. Qualitative findings supported feasibility and acceptability, but the very high attrition limits conclusions about effectiveness. |
| Glasser et al., 2016 (Israel) | Prospective single-group pre-post intervention | N = 160 pregnant women screened using the EPDS; 19 (11.9%) scored ≥10 antenatally. Nurses provided supportive counseling to 40 women. | Antenatal screening with follow-up at 6 weeks postpartum. | Non-directive supportive counseling delivered by trained nurses | None | Antenatal depressive symptoms assessed using the Edinburgh Postnatal Depression Scale | EPDS scores decreased from antenatal assessment to 6 weeks postpartum, with a greater decrease among women whose antenatal EPDS score was ≥10. The single-group design provides preliminary evidence relevant to service-level feasibility but does not establish comparative efficacy. |
| Mirghafourvand et al., 2016 (Iran) | Three-arm parallel randomized clinical trial | N = 168 Muslim pregnant women aged 15–45 years at 25–28 weeks’ gestation in their first or second pregnancy. Women with EPDS scores ≥12 were excluded and referred for mental health care. | Enrolled at 25–28 weeks’ gestation and followed through delivery. | Holy Qur’an listening with Persian translation or without translation. The recording was played for 20 minutes once weekly at the health center for 3 weeks, with daily home listening on the remaining days. | Routine pregnancy care | Preterm delivery, cesarean delivery, gestational age at birth, and neonatal anthropometric indices | Preterm delivery and cesarean delivery were numerically less frequent in both Qur’an-listening groups than in the control group, but the differences were not statistically significant. Neonatal weight, length, and head circumference also did not differ significantly between groups. |
| Jabbari et al., 2020 (Iran) | Three-arm parallel randomized controlled trial | N = 168 Muslim pregnant women aged 15–45 years at 25–28 weeks’ gestation in their first or second pregnancy. Women with EPDS scores ≥12 were excluded and referred for mental health care. Depressive symptoms were assessed using the EPDS, perceived stress using the PSS, and state and trait anxiety using the STAI. | Enrolled at 25–28 weeks’ gestation; outcomes assessed at baseline and 4 and 8 weeks after the start of the intervention. | Holy Qur’an listening with Persian translation or without translation. The recording was played for 20 minutes once weekly at the health center for 3 weeks, with daily home listening on the remaining days. | Routine pregnancy care | Depressive symptoms, perceived stress, state anxiety, and trait anxiety | Both Qur’an-listening groups had significantly lower EPDS, perceived-stress, state-anxiety, and trait-anxiety scores than the control group at 4- and 8-week follow-up. No statistically significant differences were found between the two Qur’an-listening groups. |
| Aslami et al., 2017 (Iran) | Quasi-experimental three-group comparison | Pregnant women at 16–32 weeks’ gestation with elevated Beck Depression Inventory scores (>14) and/or elevated anxiety scores (>7). The publication reports small intervention subgroups and overlapping anxiety/depression strata rather than a diagnosed MDD sample. | 16–32 weeks of gestation | Mindfulness based on Islamic-spiritual schemes for 8 weeks; group CBT for 12 weeks; sessions lasted up to 2 hours. | Control | Antenatal depressive symptoms | Both the Islamic-spiritual mindfulness program and group CBT reduced depressive symptoms compared with control, with a larger reported reduction in the Islamic-spiritual mindfulness group. The small and complex group structure limits precision. |
| Hankin et al., 2023 (USA) | Evaluator-blinded randomized clinical trial | N = 234 pregnant individuals aged 18–45 years at ≤25 weeks’ gestation with EPDS scores ≥10; current major depressive disorder was assessed using the Structured Clinical Interview for DSM-5. | Baseline at a mean of 16.7 gestational weeks, with repeated assessments through term. | Engagement session plus eight active 50-minute sessions of brief IPT delivered during pregnancy. | Enhanced usual care including engagement and maternity support services. | Antenatal depressive symptoms and major depressive disorder | Brief IPT produced faster reductions in SCL-20 and EPDS scores and fewer major depressive disorder diagnoses at the end of gestation than enhanced usual care. |
| Hassdenteufel et al., 2023 (Germany; multicenter) | Multicenter randomized controlled trial | Of 5,299 pregnant women screened, 1,153 scored >9 on the EPDS and 460 were included in the randomized trial. | Intervention delivered between 29 and 36 weeks’ gestation, with follow-up to 5 months postpartum. | Supervised electronic mindfulness-based intervention delivered over 8 weeks. | Control group receiving usual care. | General depressive symptoms, general anxiety, pregnancy- and birth-related anxiety, mindfulness, and postpartum mental health risk. | No significant intervention-by-time effects were found for general depressive symptoms or anxiety. Pregnancy- and birth-related anxiety decreased, mindfulness improved, and the proportion at risk for an adverse mental health outcome at 6 weeks postpartum was lower in the intervention group. |
| Hulsbosch et al., 2023 (Netherlands) | Randomized controlled trial | N = 219 pregnant women with elevated distress; 109 were randomized to self-guided online mindfulness and 110 to care as usual. Depressive symptoms were assessed using the Edinburgh Depression Scale, with increased symptoms defined as ≥8; pregnancy-specific anxiety was assessed using the negative-affect subscale of the Tilburg Pregnancy Distress Scale. Both instruments had established validity in Dutch pregnant women. | Antenatal period from baseline to post-program | Eight-week self-guided online mindfulness program | Care as usual | Pregnancy distress, comprising depressive symptoms and pregnancy-specific anxiety, as the primary outcome; mindfulness-related outcomes were secondary. | Pregnancy distress decreased in both groups, but no significant between-group advantage was found. Low adherence indicated potential engagement limitations for unguided delivery. |
| Leng et al., 2023 (China) | Randomized controlled trial | N = 75 adult pregnant women with heightened distress; 38 were randomized to the mobile-delivered mindfulness intervention and 37 to a web-based perinatal education program. Depression was measured using the EPDS. | Mean gestational age at baseline was 18.8 weeks; outcomes were assessed post-intervention, at 37 weeks’ gestation, and at 4–6 weeks postpartum. | Mobile-delivered four-immeasurable mindfulness-based intervention. | Web-based perinatal education program. | Perinatal depressive symptoms, mindfulness-related outcomes, and obstetric and neonatal outcomes. | The mindfulness group showed greater reductions in EPDS scores from baseline to post-intervention, sustained to 4–6 weeks postpartum, than the education control. The study also reported a lower emergency cesarean risk and higher neonatal Apgar scores; dropout was 13.2%. |
| Zhou et al., 2025 (China) | Randomized controlled trial | N = 120 women with twin pregnancies randomized to MGT or control; 109 completed the intervention. Depression was assessed using the EPDS, anxiety using the State Anxiety Inventory, stress using the Perceived Stress Scale, and mindfulness using the Five Facet Mindfulness Questionnaire. No depression eligibility threshold was reported. | Baseline during pregnancy, post-intervention during pregnancy, and 1 month postpartum | Therapist-led online mindfulness group therapy; six weekly 120-minute video-group sessions | Usual perinatal care plus health education | Perinatal depression, anxiety, perceived stress, mindfulness, and maternal and neonatal outcomes. | MGT prevented worsening of depressive symptoms, reduced perceived stress, and improved mindfulness at post-intervention and 1 month postpartum. Anxiety did not differ significantly between groups. Intention-to-treat analysis supported an effect on postpartum depressive symptoms, and low birth weight was less frequent in the intervention group. |
| Jiménez-Barragán et al., 2025 (Spain) | Randomized controlled trial | N = 70 pregnant women aged ≥18 years at 12–14 weeks’ gestation with moderate anxiety and depressive symptoms, defined by EPDS scores of 9–12; participants were randomized 1:1 to IVR or standard care. Depression was assessed using the EPDS and anxiety using the State-Trait Anxiety Inventory. | 12–14 weeks’ gestation at enrollment; 6-week intervention with post-intervention assessment. | Daily 14-minute IVR mindfulness and relaxation sessions for 6 weeks. | Standard care without virtual reality | Antenatal depressive and anxiety symptoms | The IVR group showed substantial reductions in EPDS and State-Trait Anxiety Inventory scores compared with standard care. Adherence was high, with 79% completing at least 30 sessions, and 87% reported being very satisfied, supporting preliminary feasibility and acceptability. |
| Mahoney et al., 2023 (Australia) | Prospective single-group pre-post effectiveness study | Adult Australian users of a routine-care pregnancy iCBT program, including participants with subthreshold through clinically elevated anxiety or depressive symptoms; depression was assessed using the EPDS. Gestational age was not recorded. | Pregnancy program; gestational timing was not recorded. | Three online CBT lessons completed in clinician-guided or self-guided format. | None | Antenatal depressive symptoms | Approximately 35% completed all lessons. Medium within-group reductions in anxiety and depressive symptoms were observed, but the uncontrolled design precluded causal or comparative conclusions. |
| Bhamani et al., 2024 (Pakistan) | Randomized controlled trial | N = 200 married pregnant women aged ≥18 years at 12–30 weeks’ gestation; participants with diagnosed mental or physical illness were excluded. Depressive symptoms were assessed using the EPDS, with reported Urdu psychometric support; elevated depression was not an eligibility requirement. | 12–30 weeks’ gestation; baseline and 6-week post-intervention assessment. | SM-ART delivered by trained community midwives in six weekly group sessions lasting 60–90 minutes. | Standard antenatal care; both groups also received mental health information and referral resources. | Antenatal depressive symptoms and maternal psychosocial well-being | The intervention group had higher resilience and lower depressive-symptom scores than control after 6 weeks; the adjusted decrease in depression was 1.73 points greater. Only 45/100 intervention participants attended all six sessions, and 154/200 completed post-assessment. |
| Badil et al., 2025 (Pakistan) | Controlled nonrandomized feasibility trial with a qualitative adaptation phase | N = 84 pregnant women in the first or second trimester with singleton pregnancies, male-child preference, EPDS scores ≥10, and perceived stress; 42 were assigned to intervention and 42 to control. The EPDS and PSS-10 were used. | First or second trimester; baseline, post-intervention, and follow-up assessments. | Modified Mindfulness Training Program adapted using the ADAPT-ITT framework and delivered by a trained female facilitator. | Routine hospital care without the mindfulness intervention. | Edinburgh Postnatal Depression Scale scores and perceived stress | Depression and perceived-stress scores decreased significantly over time in the intervention group, while no significant changes occurred in the control group. The study provides preliminary feasibility and within-study comparative signals but does not establish definitive efficacy or scalability. |

**Note.** Depression or distress measures, diagnostic criteria, eligibility thresholds, gestational timing, and validation information are reported from the primary publications where available. Where a study enrolled a general or prevention-oriented antenatal sample rather than requiring elevated depressive symptoms, this is stated explicitly. Mirghafourvand et al. (2016) and Jabbari et al. (2020) reported different outcome domains from closely related randomized trial procedures; each publication is presented separately according to the outcomes reported in that article.

**Abbreviations:** CBT, cognitive behavioral therapy; IPT, interpersonal psychotherapy; EPDS, Edinburgh Postnatal Depression Scale; MDD, major depressive disorder; ANC, antenatal care; eMBI, electronic mindfulness-based intervention; IVR, immersive virtual reality; SM-ART, Safe Motherhood–Accessible Resilience Training.
