# Supplementary material for: A Narrative Review of Implementing Nonpharmacological Interventions for Antenatal Depression in Low Resource Settings With Implications for Palestine
Source: Depress Anxiety. 2026 Aug 11;2026:4692841. doi: 10.1155/da/4692841 (PMC13462660; doi:10.1155/da/4692841)
Supplement: Supplementary file 3 [file DA-2026-4692841-s003.docx]

**Supplementary Table S2.** *Clinical effects, delivery characteristics, and implementation considerations across intervention categories*

| Intervention category | Included studies and context | Clinical findings | Dose and delivery format | Provider and service setting | Reported feasibility indicators | Main implementation gaps | Relevance to Palestine and similar settings |
| --- | --- | --- | --- | --- | --- | --- | --- |
| Cognitive behavioral therapy, interpersonal psychotherapy and related psychotherapies | Jesse et al. 2015, United States; Burns et al. 2013, United Kingdom; Mehri et al. 2019, Iran; Rahman et al. 2008, rural Pakistan; Spinelli and Endicott 2003, United States; Spinelli 1997, United States; Hankin et al. 2023, United States obstetrics and gynecology clinics; Mahoney et al. 2023, Australian routine internet-delivered care | Randomized and controlled studies generally reported greater reductions in antenatal depressive symptoms than usual care or active comparators. Brief IPT also reduced MDD diagnoses by term. Uncontrolled and routine-care studies reported within-group improvement but provided preliminary rather than causal evidence. | Programs ranged from 6 weeks to 16 sessions and included brief clinic-based, individual, group, task-shared, and internet-delivered formats. The Rahman program extended from late pregnancy through 12 months postpartum. | Delivered by trained therapists, primary health workers, or digital platforms within antenatal, obstetric, public health, and primary care settings. | Reported participation and completion, integration into routine services, brief and group formats, supervised task sharing, and internet-supported delivery provided preliminary service-level feasibility indicators. | Formal measures of acceptability, fidelity, implementation cost, organizational adoption, population reach, and long-term sustainability were rarely reported. Attrition and completion varied across studies. | This category had the strongest comparative evidence and the clearest examples of supervised task sharing. Brief and group formats may fit routine antenatal services, but implementation requires training, supervision, referral pathways, protected staff time, and monitoring of retention. |
| Depression-specific acupuncture | Manber et al. 2004 and Manber et al. 2010, United States | Depression-specific acupuncture produced greater symptom reduction than massage and, in the larger trial, than control acupuncture. The two control conditions did not differ significantly. | Structured acute treatment delivered over approximately 8 weeks; the larger trial used 12 sessions over 8 weeks. | Delivered by trained acupuncturists using protocolized point prescriptions in controlled clinical settings | The structured protocol was successfully delivered under controlled research conditions; routine-service feasibility was not evaluated. | Routine-service feasibility, practitioner availability, patient preference, implementation cost, population reach, and sustainability were not evaluated. | May be considered only as an adjunct where trained practitioners, safety procedures, supervision, and clinical governance are available. |
| Mindfulness and mind–body interventions, including digital delivery | Beattie et al. 2017; Hassdenteufel et al. 2023; Hulsbosch et al. 2023; Leng et al. 2023; Zhou et al. 2025; Jiménez-Barragán et al. 2025; Badil et al. 2025 | Findings varied by format and outcome. Selected clinic-embedded, mobile-delivered, therapist-led group, and IVR interventions reported favorable symptom findings. The Hassdenteufel eMBI trial did not show a significant effect on general depressive symptoms or anxiety, although pregnancy- and birth-related anxiety and mindfulness improved. The unguided Hulsbosch intervention showed no between-group advantage and had adherence limitations. Badil et al. reported preliminary comparative improvements but not definitive efficacy. | Formats ranged from brief visit-aligned or IVR sessions to structured programs lasting approximately 6–8 weeks. Zhou et al. used six weekly 120-minute therapist-led groups; Hulsbosch used an 8-week self-guided program; Hassdenteufel used an 8-week supervised eMBI; Leng used a mobile-delivered program; Badil used an adapted group mindfulness program. | Delivered in maternity clinics, therapist-led online groups, supervised electronic platforms, self-guided websites, mobile applications, IVR settings, and culturally adapted groups. | Integration with antenatal visits, therapist support, adherence and dropout data, group delivery, and culturally adapted delivery provided preliminary feasibility indicators. In the IVR trial, 79% of participants completed at least 30 sessions and 87% reported being very satisfied, providing direct adherence and satisfaction evidence. However, feasibility indicators were reported inconsistently across the remaining studies and did not establish scalability. | Optimal dose and the contribution of guidance were unclear. Except for selected studies reporting satisfaction or adherence, formal acceptability, fidelity, implementation cost, privacy, equitable digital access, technical support, population reach, and long-term sustainability were inconsistently assessed. | Potential adjunctive options where privacy, access, technical support, supervision, and clinical escalation procedures can be ensured. The available findings suggest that guidance and adherence support may be important, but supervised and unguided formats were not directly compared under otherwise equivalent conditions. |
| Midwife- or nurse-supported counseling | Glasser et al. 2016, antenatal care in an ultra-Orthodox Israeli community | EPDS scores decreased from antenatal assessment to 6 weeks postpartum, with greater improvement among women with elevated antenatal scores. The uncontrolled design supports preliminary feasibility rather than comparative efficacy. | Brief non-directive supportive counseling integrated into antenatal care, with follow-up at 6 weeks postpartum. | Delivered by trained nurses within routine antenatal or public health services | Service-based EPDS screening, nurse delivery, and integration into routine care provided preliminary evidence relevant to task sharing. | Comparative effectiveness, formal acceptability, fidelity, workload, supervision requirements, implementation cost, population reach, and sustainability were not evaluated. | Potentially relevant to nurse-led and midwife-led antenatal care, but controlled evaluation, supervision, workload assessment, and referral capacity are required. |
| Culturally or spiritually integrated interventions and low- and middle-income country group-skills programs | Mirghafourvand et al. 2016 and Jabbari et al. 2020, Iran; Aslami et al. 2017, Iran; Bhamani et al. 2024, Pakistan | Jabbari et al. reported lower depression, stress, and anxiety scores after Qur’an listening, whereas Mirghafourvand et al. found nonsignificant reductions in preterm and cesarean delivery and no neonatal anthropometric differences. Other culturally integrated programs also showed favorable depressive-symptom findings, although designs and populations varied; Bhamani enrolled a general antenatal sample. | Qur’an listening for 20 minutes once weekly at a health center for 3 weeks, accompanied by daily home listening; Islamic-spiritual mindfulness for 8 weeks; group CBT for 12 weeks; and SM-ART delivered in six weekly 60–90-minute group sessions. | Delivered through clinic sessions, home practice, culturally adapted groups, and programs embedded in antenatal care; SM-ART was delivered by trained community midwives. | The Qur’an-listening interventions combined brief health-center sessions with monitored home practice and had low attrition. Group delivery, home practice, routine-service integration, participation, and completion provided preliminary delivery indicators. These findings did not constitute formal evidence of broader acceptability or scalability. | The Qur’an studies recruited Muslim women who believed in the Qur’an and excluded women with EPDS scores ≥12; therefore, applicability to clinically significant depression and to women with different cultural or religious preferences remains uncertain. Formal assessment of informed choice, fidelity, implementation cost, organizational adoption, population reach, and sustainability was not reported. | Potentially relevant as an optional culturally and spiritually integrated approach for women who prefer such care. Implementation should preserve informed choice, access to non-faith-based alternatives, clinical screening, referral for elevated symptoms, and appropriate clinical oversight. |

**Note.** Clinical findings describe the direction of antenatal depressive-symptom change within each intervention category and do not represent pooled estimates. Feasibility indicators were derived from reported features such as integration into routine care, task sharing, participation, attendance, adherence, retention, or completion. These indicators should not be interpreted as formal evidence of acceptability, adoption, scalability, implementation cost, population reach, or sustainability unless these outcomes were directly assessed in the primary study.
